# Para‑testicular arteriovenous malformation: A case report and mini‑review of the literature

**DOI:** 10.3892/mi.2023.88

**Published:** 2023-06-06

**Authors:** Rawa Bapir, Fahmi H. Kakamad, Ismaeel Aghaways, Ari M. Abdullah, Marwan N. Hassan, Ayoob Asaad Mohammed Abid, Sabah Jalal Hasan, Karzan M. Salih, Hussein M. Hamasalih

**Affiliations:** 1 Department of Scientific Affairs, Smart Health Tower, University of Sulaimani, Sulaymaniyah, Kurdistan 46000, Iraq; 2 Kscien Organization for Scientific Research, Sulaymaniyah, Kurdistan 46000, Iraq; 3 Department of Urology, Sulaymaniyah Surgical Teaching Hospital, Sulaymaniyah, Kurdistan 46000, Iraq; 4 College of Medicine, University of Sulaimani, Sulaymaniyah, Kurdistan 46000, Iraq; 5 Department of Pathology, Sulaymaniyah Surgical Teaching Hospital, Sulaymaniyah, Kurdistan 46000, Iraq

**Keywords:** vascular malformation, para-testicular mass, intra-scrotal, spermatic cord

## Abstract

Arteriovenous malformations from para-testicular structures are very rare, with only a limited number of cases reported in the literature. The present study reports a rare case of para-testicular arteriovenous malformation. A 6-year-old boy presented with painless swelling in the scrotum for 6 months. Upon examination, a non-tender and non-pulsatile cystic swelling was observed in the right hemi-scrotum below the testis. A scrotal ultrasound revealed a separate cystic lesion with a normal texture and the vascularity of both testes. Under general anesthesia, via a small scrotal incision, a cystic, blood-filled mass was excised. The results of a histopathological examination were suggestive of vascular malformation. The case described in the present study aims to shed light on vascular malformations. A number of vascular malformations are incorrectly referred to as hemangiomas, and numerous patients undergo inappropriate therapy due to this misclassification. Although para-testicular arteriovenous malformation is a very rare condition, it should be included in the differential diagnosis of para-testicular lesions.

## Introduction

Arteriovenous malformations (AVMs) are vascular system anomalies considered to develop during embryogenesis, fetal development, or shortly after birth (1). AVMs are characterized by the tangling of arteries and veins without the presence of capillaries. This leads to the rapid and high-pressure blood flow through these abnormal vessels, hindering the delivery of arterial blood to the tissues. As a result, varying degrees of ischemia occur (1). AVMs are the most challenging vascular anomalies to manage and are frequently associated with morbidity and mortality (2). They arise due to developmental changes in blood vessel formation, exhibit proportional growth alongside the child's development, and are identified by the presence of enlarged feeding vessels, excessive arteriovenous connections at the nidus level, and high vascularity. While some AVMs may not present any symptoms, others can manifest as increased size, bleeding, pain, or conditions such as azoospermia, infertility, heart failure, and potentially life-threatening hemorrhages (3,4). Their most common locations are the neck, trunks, extremities, and extracranial and intracranial areas (3). The involvement of intra-scrotal components is extremely rare, generally manifesting as para-or intra-testicular masses (1). The para-testicular area contains a variety of structures, including the tunica vaginalis, lymphatic channels, ductus deferens, epididymis, vessels, spermatic cord, and other testicular suppurative tissues (5). AVMs from these structures are very rare, with only a limited number of cases of the spermatic cord or scrotal wall reported in the literature (1,4).

The present study reports an extremely rare case of para-testicular AVM without the involvement of the epididymis or spermatic cord.

## Case report

**Figure 1:**
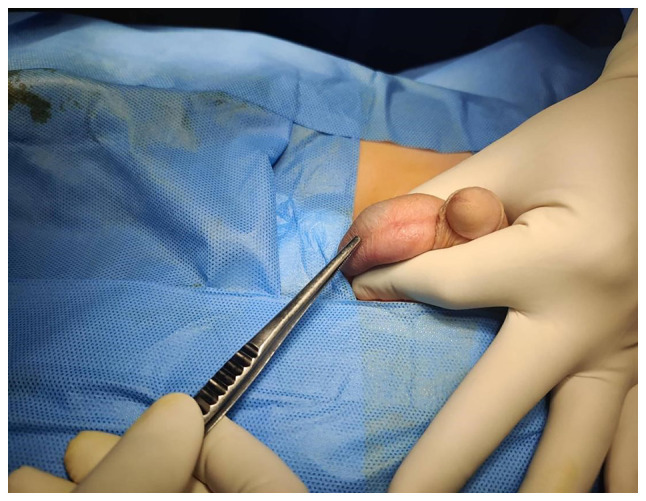
Intraoperative image illustrating a cystic mass with a blush color under the skin.

**Figure 2:**
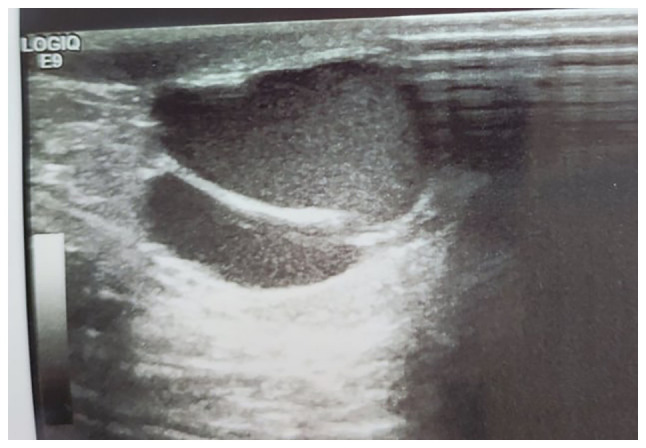
A bilocular cystic lesion measuring 17x9 mm, with homogeneous low-level internal echoes and thin septa observed in the right hemi-scrotum (para-testicular), below and separate from the right testicle; no obvious flow was observed inside the lesion.

**Figure 3:**
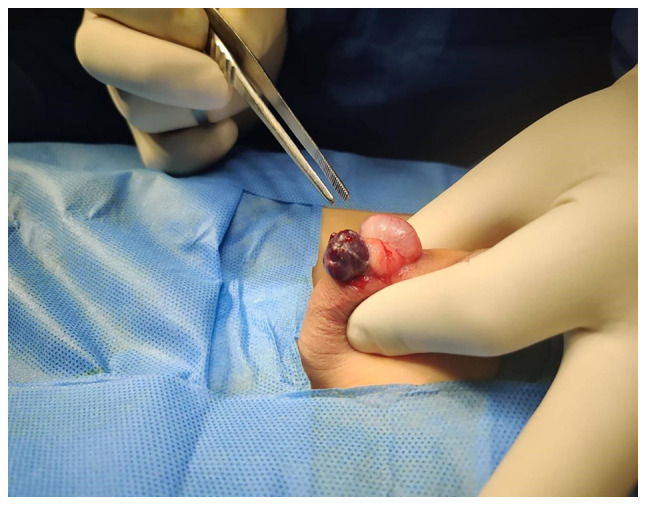
Intraoperative image illustrating a cystic blood-filled mass below the testis.

**Figure 4:**
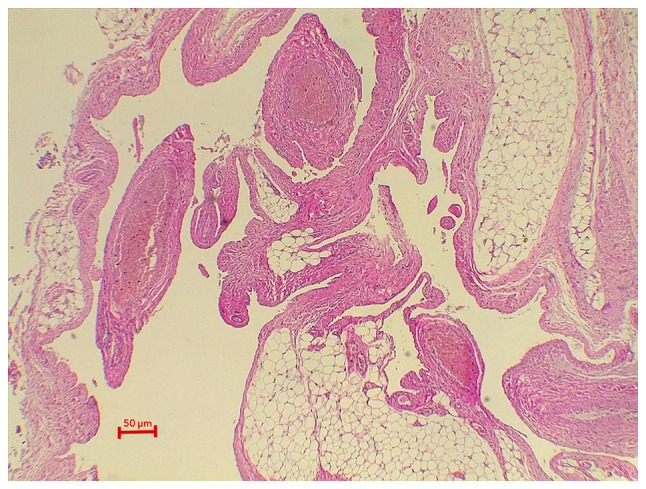
The section reveals fibrofatty tissue fragments containing irregular, different size, branching vascular spaces that are lined by endothelial cells with mature adipose tissue (magnification, x400).

A 6-year-old boy presented with a painless swelling on the right side of the scrotum that his parents had observed for 6 months. There was no history of surgery or trauma. Upon an examination, a blush-colored, non-tender, immobile, and non-pulsatile cystic swelling was observed in the right hemi-scrotum below the testis (Fig. 1). A scrotal Doppler ultrasound (U/S) revealed a separate 20x12 mm bilocular cystic lesion below the right testis with a normal texture, and the vascularity of both testes (Fig. 2). Under general anesthesia, via a small scrotal incision, the surgery was performed. A cystic, blood-filled mass was found and excised (Fig. 3). Intraoperatively, there were no complications. The patient was discharged the same day, and his post-operative period was uneventful. A histopathological examination was performed under the following conditions: The sections (5 µm-thick) were paraffin-embedded and fixed with 10% neutral-buffered formalin at room temperature for 24 h. The sections were then stained with hematoxylin and eosin (Bio Optica Co.) for 1-2 min at room temperature and examined under a light microscope (Leica Microsystems GmbH). Histopathological examinations also revealed fibrofatty tissue fragments with irregular different-sized branching vascular spaces lined by endothelial cells (Fig. 4). The result was consistent with a vascular malformation.

## Discussion

Scrotal swelling is a relatively frequent medical condition. Space-occupying lesions from these sites may be neoplastic or non-neoplastic (3). Neoplastic lesions can be benign or cancerous. Non-neoplastic masses include inflammation, epididymal cysts, spermatic cord cysts, spermatoceles, hydroceles, pyoceles, and hernia (5-7). Approximately 5% of all intra-scrotal masses are para-testicular neoplasms and the epididymis accounts for 20-30% of these (8). The spermatic cord is responsible for 70% of all lesions, with lipomas being the most common. The most frequent epididymis tumors are adenomatoid tumors, followed by leiomyomas. Other benign tumors include fibroma, neurofibroma, hemangioma, and papillary cystadenoma (7).

Although vascular lesions, such as varicocele, hemangioma, lymphangioma, and AVMs are possible, they are uncommon and are rarely described in the medical literature (9). Adult males frequently develop benign vascular lesions. Varicoceles are the most frequent lesion, whereas AVMs are the rarest (3). Vascular malformations are collections of aberrant vessels detected at birth in 90% of cases (10). These lesions develop alongside the infant and exhibit no signs of endothelial growth (10). AVM is well-known due to its presence in the central nervous system, although it can be present everywhere (1). The spermatic cord and scrotal wall are the most commonly reported sites for scrotal or intra-scrotal AVMs (1,5,11-13). AVMs of the spermatic cord are benign lesions comprised of complicated tangles of swollen, dilated arteries and veins with no intervening capillaries (1). In this case, the para-testicular AVM is independent and unattached to the surrounding structure (spermatic cord or epididymis).

Mulliken and Glowacki (14) categorized vascular abnormalities as vascular tumors (infantile hemangioma, kaposiform hemangioendothelioma, congenital hemangioma, and tufted angioma) and vascular malformations (AVM, lymphatic malformation, venous malformation, and capillary malformation). In the medical literature, a number of vascular malformations were incorrectly referred to as hemangiomas, and numerous patients have undergone inappropriate therapy due to this misclassification (15). The majority of patients are asymptomatic and present with a slow-growing, non-tender mass. A rapidly expanding, non-tender mass is rarely reported by some patients (7). Upon examination, they appear as masses with dilated vessels overlying them and a thrill (16). However, Kang *et al* (17) reported a case of para-testicular AVM with a painful gradual enlargement of the left hemiscrotum. The case presented herein exhibited scrotal swelling for 6 months without any pain or tenderness. Upon examination, it appeared as a blushing mass under the skin. There was no thrill on palpation. Pre-operatively, it was suspected to be a hemangioma.

U/S is the preferred initial examination, since it is readily available, inexpensive, and is associated with excellent sensitivity and specificity. It is used to determine whether a lesion is benign or malignant, delineates borders, and defines echogenicity, vascularity, invasive behavior, and neighboring tissues. If a U/S indicates a well-bordered, isolated, homogeneous, non-invasive lesion, the use of contrast-enhanced computed tomography or magnetic resonance imaging (MRI) may be limited. If the results of the U/S are ambiguous or dubious, further radiography can be conducted using computed tomography or MRI, and tumor markers for testicular cancer can be sent for assessment (18). A U/S can distinguish between intratesticular and extra-testicular lesions, as well as solid and cystic lesions, with 90-100% accuracy. This difference is critical as the majority of para-testicular masses are benign, whereas the majority of testicular masses are cancerous (19). A U/S usually reveals a network of numerous vascular channels, which may resemble a varicocele (16). As embolization may be performed concurrently, angiography is the gold standard for evaluating arteriovenous malformations (20). The U/S of the case in the present study revealed a separate bilocular cystic lesion below the right testis with normal texture and vascularity of both testes.

The preferred therapeutic options for AVMs are sclerotherapy, embolization, and surgical excision (4). Sclerotherapy reduces the size of the venous nidus prior to surgical excision, and embolization eases the resection process with the least amount of bleeding (13). Finally, surgery is the only effective and approved therapy (13). Some consequences may occur as abnormalities are often long and poorly defined. There is a risk of acute bleeding, and poor procedure care may result in impotence and infertility (10,13). The case described herein underwent surgical resection. There were no intraoperative complications.

In conclusion, para-testicular AVMs are a very rare condition. Based on this case, the authors suggest that AVM should be included in the differential diagnosis of para-testicular lesions.

## Data Availability

The datasets used and/or analyzed during the current study are available from the corresponding author on reasonable request.
